# AI assistance in enterprise UX design workflows: enhancing design brief creation for designers

**DOI:** 10.3389/frai.2024.1404647

**Published:** 2024-11-12

**Authors:** Zijian Zhu, Hyemin Lee, Younghwan Pan, Pengyu Cai

**Affiliations:** Interaction Design Lab., Graduate School of Techno Design, Kookmin University, Seoul, Republic of Korea

**Keywords:** design brief, AI thinking, generative AI, design management, user experience

## Abstract

The study explores the impact of AI tools on the daily tasks of designers in corporate environments, with a focus on the creation and evaluation processes of design briefs. Given ChatGPT’s advanced natural language processing capabilities and its potential to meet the complex communication and analysis needs of design work, this tool was selected to investigate its application in designers’ workflows. Through expert interviews, experimental testing, and third-party expert evaluations, we collected and analyzed data to understand the impact of AI on work processes. The findings indicate that AI tools significantly enhance both operational experience and subjective perceptions across most tasks. Additionally, the study provides a visual comparison of the testing process through a user experience map, highlighting AI’s positive influence on work efficiency, information retrieval, verification, analysis, communication, and decision-making. However, challenges remain in ensuring information authenticity, protecting content copyright, and maintaining professional identity. The primary objective is to gain a comprehensive understanding of the current state of AI application in business contexts and its impact on designers’ roles. By analyzing real-world feedback, the research aims to identify the strengths and weaknesses of AI solutions in enterprises and offer practical recommendations. The study underscores the importance of integrating AI thinking into workflows and adopting a human-centric approach for the future development of corporate work environments.

## 1 Introduction

Generative AI, as a new productivity tool, has already been widely applied in enterprises. According to OpenAI’s market research, as of March 2023, at least 10% of job tasks may be affected, with approximately 19% of workers potentially experiencing more than a 50% change in their tasks (Eloundou et al., 2023). Although the research report does not directly address the impact of AI language model applications like “ChatGPT” on design professions, the advancements in data and computational power are transforming traditional work patterns. Studies have shown that integrating AI thinking into projects can enhance design efficiency and innovation, while also improving product user experience (Al-Sadi and Miller, 2023).

In efficiency-driven business projects, developing detailed execution plans is crucial, as this can reduce redundant work for designers in later stages and save costs. From both academic (Zhang et al., 2024) and practical perspectives, organizations have established standardized processes to address most production issues. However, due to the dynamic nature of projects, unpredictable situations still arise, such as poor information transmission, changing client requirements, and time and cost pressures (Petersen, 2010; Tang et al., 2005; Cooper, 1998; Petersen, 2009). Additionally, designers may face challenges in dealing with projects in unfamiliar fields (such as healthcare, architecture, and finance) due to a lack of background knowledge. Addressing these issues typically requires designers to have extensive design management experience.

In a project’s regular workflow, the design brief is a critical tool for organizing design resources and requirements (Zhang et al., 2024). It is used in the initial stages of a project (Jevnaker, 2005; Petersen and Phillips, 2011) to reflect the preliminary ideas of clients and stakeholders and is further developed through interaction (Paton and Dorst, 2011). Creating a design brief requires designers to invest substantial time and effort in research and organization. However, current AI language models possess general knowledge capabilities that can help designers overcome cross-disciplinary knowledge gaps. By configuring AI as a designer to interpret requirements from different fields and propose solutions, the efficiency and accuracy of designers working in unfamiliar areas can be significantly improved.

Although there has been extensive research on the application of AI tools in design work (Olga et al., 2023; Jeong, 2023; Singh et al., 2023; Fui-Hoon Nah et al., 2023), most studies employ big data quantitative analysis methods (Sano and Yamada, 2022; Cico et al., 2023; Cheng et al., 2022; Nauta et al., 2022; Shi et al., 2023) and lack detailed descriptions of specific work scenarios and actual processes. This indicates that the academic community is still in the early stages of defining and exploring the patterns of AI-assisted work. Particularly in the field of design brief creation, there is relatively little research, especially on the impact of AI tools on designers’ and clients’ perceptions and behaviors.

This paper will explore the following questions:

RQ1: What common challenges and issues do designers face when creating design briefs in their daily work?

RQ2: What elements do designers focus on when using ChatGPT to create design briefs?

RQ3: What differences exist in efficiency, cost, accuracy, and client satisfaction between using ChatGPT and traditional methods to create design briefs?

RQ4: What are the benefits and limitations of using AI tools in workflows at the enterprise level?

This study expands the theoretical framework for the feasibility of applying AI in design projects, exploring the impact of AI tools on designers during the creation of design briefs. It advances the understanding of AI-assisted workflows and enriches the theoretical discussion on the interaction between designers and AI tools, providing a foundation for future research. Additionally, we examine the factors influencing designers’ willingness to use these tools, aiming to help industry decision-makers and developers enhance the experience and effectiveness of these tools. By extending the academic discourse on AI tools in the design field, we offer valuable insights to scholars, industry experts, and policymakers, thereby promoting the continuous advancement of AI technology in design and leading sustainable development.

From an innovation perspective, we use user experience maps to thoroughly explore the application of ChatGPT in the entire process of creating design briefs, optimizing the existing framework. Integrating AI thinking into the workflow of design brief creation aims to help designers effectively use AI tools, significantly enhancing work efficiency and design quality. This provides innovative solutions for implementing design projects within enterprises.

## 2 Literature review

### 2.1 The applications of GenAI

Generative Artificial Intelligence (GenAI) is a rapidly evolving subfield of Artificial Intelligence that utilizes advanced models such as Generative Adversarial Networks (GANs), Variational Autoencoders (VAEs), and Transformer architectures to produce high-quality and diverse content (Zhang, 2024; Rohan, 2024). Despite being widely regarded as a “fashionable technology” in the past year, it is essential to understand its uniqueness and associated risks, including bias, transparency, hallucinations, misuse, and societal impact. Responsible integration of this technology requires understanding its advantages and disadvantages and implementing appropriate countermeasures (Banh and Strobel, 2023).

Over the past year, GenAI has been validated in the market and has spawned numerous application scenarios. In content creation, it can be used for writing articles, artistic creation, and music generation. In decision support, GenAI can power intelligent question-and-answer systems, supporting tasks such as IT help desks, cooking recipe suggestions, and medical advice. Additionally, GenAI has shown significant potential in commercial applications, such as automatically generating SEO content, code generation, and automated customer service (Feuerriegel et al., 2024). Its applications extend to smart publishing, advertising content creation, and the financial sector, where its ability to generate unique content is transforming professional workflows and demonstrating considerable potential as a knowledge assistant (Cevallos et al., 2023). As GenAI becomes more deeply integrated into various industries and daily life, it is crucial to take proactive measures to ensure its ethical, safe, and fair use, thereby upholding societal values and norms amid rapid technological innovation (Shang, 2024).

To better understand and apply GenAI, Strobel further defined five types of generative AI: Generators, Reimaginators, Synthesizers, Assistants, and Enablers (Strobel et al., 2024). Generators create new content based on user input; reimaginators reinterpret data, altering its form while maintaining semantic stability; synthesizers generate data for AI model training or IT testing; assistants provide domain-specific support; and enablers offer infrastructure support. This study primarily focuses on the roles of Generators and Assistants in the entire business planning process, including proposal writing, product concept output, detailed design, front-and back-end code editing, product implementation, and marketing (Ghimire et al., 2024). These elements are critical considerations when creating a design brief.

### 2.2 AI integration in design workflows

In large-scale projects, design briefs are typically led by product design managers with extensive experience and comprehensive skills, who can effectively manage teams and control costs. To explore how AI can assist these experts, we must first identify the specific applications and potential of AI in the design field.

Within enterprises, AI positively impacts management theories, including decision-making, knowledge management, customer service, human resource management, and administrative tasks (Likert, 1932). In the design domain, the influence of generative AI is expanding, from text and image generation to predicting user behavior and optimizing design decisions (Zhu and Luo, 2022). Generative AI is transforming the way UX designers work and profoundly affecting workflows.

Our preliminary research reveals two main perspectives among company designers regarding AI. Some say, “Great, my work efficiency has significantly improved, allowing me to focus on creativity and quickly generate outputs.” Others express concern, saying, “AI is replacing my job, and I might become unemployed.” From a management perspective, they aim for their teams to complete tasks efficiently and cost-effectively. They are eager to adopt new productivity tools and refer to this adoption as a “revolution”.

In his 2017 study, Girling (2017) predicted that artificial intelligence would have a profound impact on the design field. He suggested that as design technology advances, the learning curve will significantly decrease, making it possible for anyone to become a designer. Additionally, he predicted that the role of designers would shift from creators to curators. These predictions appear highly foresighted today.

The development of AI technology has greatly expanded the capabilities of designers. In the past, designers focused on 2D graphic design needed extensive time to learn 3D modeling software and set parameters to create models. Now, they can quickly generate 3D models by simply conveying their ideas through text prompts to AI. This shift has substantially lowered the barrier from 2D to 3D design, making the design process more convenient and efficient.

However, despite the new tools and possibilities provided by AI technology, Bertão and Joo’s (2021) study indicates that designers currently prefer to view AI as an assistant to enhance workflow efficiency, rather than as a creative partner. The impact of AI is primarily concentrated in fields such as UX/UI design, art, industrial design, graphic design, and architectural design. Nonetheless, this does not mean that other design fields, such as fashion design and architectural design, will remain unaffected by AI. In fact, as algorithms and data evolve, it is foreseeable that AI will influence a broader range of design areas (see Table 1).

**Table 1:** The impact of GenAI on design fields.

Domain	Impact on designers	Reference
UX/UI	Create scenarios, assist decision-making, improve efficiency	Bertão and Joo (2021)
Fine art	Inspire creativity, new expressions, and work with machines	DiPaola et al. (2018) and Mukherjee Chakraborty (2023)
Graphic design	Role transformation, efficiency improvement, innovation and experimentation, customization	Forrester (2023) and Shevde (2023)
Industrial design	Quickly generate and evaluate many options, improve innovation and efficiency	RedBlink (2023)

Using a design brief is a comprehensive way to understand a project, and AI plays a crucial role in several key areas, including cost control, schedule optimization, quality control, sustainability assessment, and visualization. Below is a summary of relevant literature (see Table 2).

**Table 2:** The impact of GenAI on design project.

Workflow	The impact of GenAI on project workflows	Reference
Cost control	Budget forecast	Ghimire et al. (2023)
	AI and Intelligent vision applied to architecture and construction	Baduge et al. (2022)
	Optimized machine learning modeling is used for cost and duration prediction of tunnel projects	Mahmoodzadeh et al. (2022)
Schedule optimization	Optimized machine learning modeling is used for cost and duration prediction of tunnel projects	Abioye et al. (2021)
	Machine learning in architecture from shallow to deep learning	Xu et al. (2021)
Quality control	Machine learning methods for improving highway construction quality	Saravanan et al. (2018)
Sustainability	AI’s impact on design sustainability	Kar et al. (2022)
	AI in green building design	Debrah et al. (2022)
	Machine learning applications in energy performance prediction of urban buildings	Fathi et al. (2020)
visualization	Reasoning about drawing elements and space usage in drawings using semantic segmentation	Seo et al. (2020)

### 2.3 Design briefs within the workflow

The design brief, as a critical tool for project initiation, functions as a bridge between business and design (Jevnaker, 2005). It helps designers and clients reach a consensus, clarifying the project’s goals and expected outcomes (Paton and Dorst, 2011). The importance of the design brief lies not only in providing a collaborative framework but also in defining task responsibilities, organizing resources, tracking key project milestones, and outlining the steps to be taken, ensuring that resources and processes are well-documented. Cross-functional collaboration is essential in product design. Parkman and Malkewitz (2019) further emphasize the importance of design briefs, considering them as outputs of knowledge-based cross-functional collaboration (Parkman and Malkewitz, 2019).

The core functions of a design brief are primarily threefold:

1. Assessment: the design brief serves as an evaluation tool at the project’s inception, reflecting the needs of stakeholders.
2. Agreement: it acts as an agreement between the client and the designer, and a key tool for validating the integration of business and innovation at critical project milestones.
3. Documentation: as a working document for iterative development, the design brief guides the project continuously until completion.

Although the format of the design brief may vary by organization, the underlying logic remains consistent. The design brief should cover three phases: the initial stage, the ongoing stage, and the final stage, with each phase authored by different stakeholders (Rudin, 2019). Clients are responsible for defining the needs and objectives during the initiation phase. Based on this, the design team develops the brief, proposing constraints, cost alternatives, and validating execution plans. Once the design brief is completed, key milestones must be confirmed with the client to minimize deviations during project execution.

In book “Creating the Perfect Design Brief,” Philip proposed a widely accepted template that outlines seven key elements: “Project Overview and Background, Category Review, Target Audience Review, Company Portfolio, Business Objectives and Design Strategy, Project Scope, Timeline, and Budget, and Research Data & Appendix.” Similarly, Stone in 2010 presented a framework comprising ten elements: “Background Summary, Overview, Driver, Audience, Competitors, Tone, Message, Visuals, Details, and People” as headings for the structure and content (Stone, 2010). Regardless of the framework used, the core objective is to serve as a strategic manual guiding project success.

In addition to creating content guidelines, we also need an evaluation framework for the design brief. Here, we reference the Design Quality Criteria (DQC) framework (Petersen and Phillips, 2011; Petersen and Joo, 2015) as the evaluation model for the design brief outputs. The evaluation model includes three dimensions: strategy, content, and performance. The strategy dimension covers the company’s philosophy (history, values, vision, mission), structure (sectors, business model, competitive advantage), and innovation. The content dimension addresses social aspects (consumer needs and activities), environmental considerations, and economic feasibility. The performance dimension focuses on processes (budget and timeline), functionality (unique selling points), and product aesthetics.

The structure of a design brief can vary depending on the project’s nature and complexity (Phillips, 2012). Due to the diversity of projects and the broad range of knowledge areas involved, Jones and Askland proposed seven key elements to ensure comprehensiveness in creation: (a). Project Overview; (b). Market Background and Competitive Analysis; (c). Target Audience Assessment; (d). Company Portfolio; (e). Business Objectives and Design Strategy; (f). Project Scope; (g). Research Data and Appendix (Chesbrough and Crowther, 2006). These requirements clearly illustrate the high demands on designers’ capabilities in creating design briefs. Petersen (2010) also noted potential issues during the creation process, such as a lack of genuine business logic, insufficient understanding of design, and a disconnect with budget considerations. Designers often need to invest significant time to bridge the cognitive gap with client (Dewulf, 2020). GenAI, with its vast data sources and advanced neural networks, has demonstrated excellent “knowledge transfer” capabilities (Ritala et al., 2023). This can be particularly useful in addressing the issue of “cross-domain knowledge” during the creation of design briefs.

## 3 Methodology

On our preliminary research, we explored the feasibility of using artificial intelligence (AI) technology to create design briefs. This paper aims to analyze and validate this process through qualitative research methods. Qualitative research can deeply reveal the motivations and emotions behind the study subject and allows for flexible adjustments to the research strategy based on real-time findings (Kim et al., 2017). We conducted in-depth interviews to document participants’ behaviors and thought processes when using AI tools to create design briefs. Using user experience maps for visual comparison, we aimed to uncover underlying perspectives and behavior patterns. Through this approach, we hope to gain a clear understanding of the effectiveness and value of ChatGPT in the creation of design briefs.

### 3.1 Experiment design

#### 3.1.1 Participants

The participants of this study are primarily drawn from emerging fields such as digital product and interaction design, as these areas are often the first to adopt new technologies (Chesbrough and Crowther, 2006). To ensure the accuracy and representativeness of the research results, we recruited 8 senior UX design experts with over five years of work experience across various product lines through the company’s internal network. These experts not only possess a deep understanding of industry trends and design processes but also have extensive project experience, particularly in addressing challenges related to unclear objectives and incomplete requirement documentation. Their involvement significantly enhances the depth and reliability of this study.

Additionally, user experience designers, with their profound understanding of user behavior, interaction processes, and product innovation, are ideal candidates for researching the application of new technologies in the design field. Designers must thoroughly grasp the relationship between customer needs, user behavior, and business goals to create products that better meet user demands, further highlighting their crucial role in this study.

#### 3.1.2 Study process and plan

The experiment is divided into two phases to understand designers’ behaviors and experiences in creating design briefs. Initially, through semi-structured interviews and specific design tasks, we aim to understand the designers’ routine operations and challenges, focusing on key task nodes, touchpoints, and pain points. This phase explores research question 1 and visualizes the data. Subsequently, AI tools like ChatGPT are introduced. Designers are given set tasks, and their performance using AI tools is observed and recorded. Follow-up interviews are conducted to delve deeper into research questions 2, 3, and 4. This approach allows us to compare and analyze the differences in outcomes when designers use AI tools versus when they do not.

Considering the constraints of qualitative research methods, such as the testing environment and the number of participants, we adopted a within-subject design to ensure internal consistency and reduce potential bias. To minimize order effects, such as fatigue impacting the experimental results, the experiment for each participant is divided into two phases. The initial phase involves a 30-min in-depth interview to ease the participants’ psychological burden. In the second phase, participants perform routine design brief creation in a preset virtual project environment, with the duration controlled within one hour, noting completion status and time.

**Figure 1:**
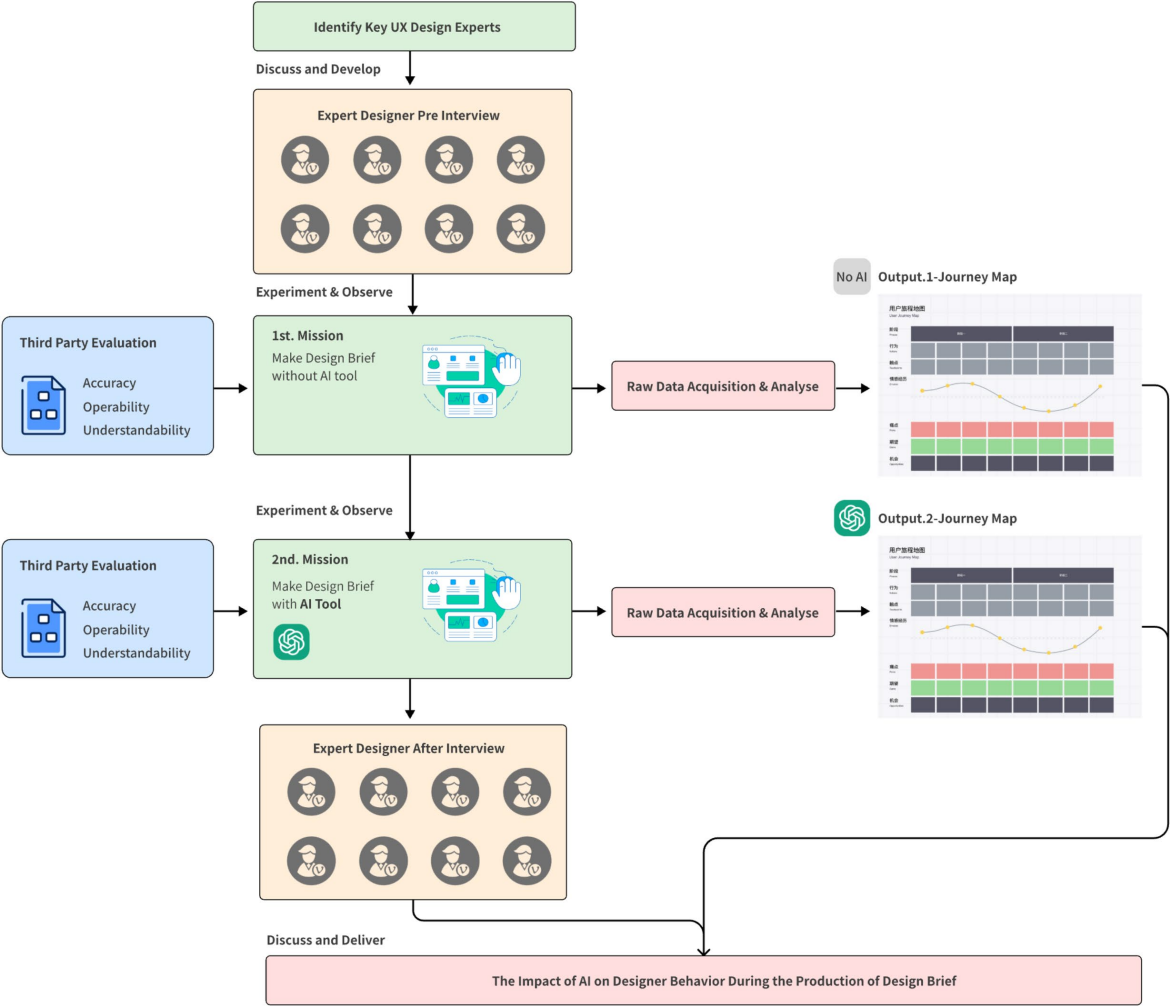
Experimental process.

After completing the initial experiment, participants are invited back a week later to use AI tools to assist in creating the design brief. During task execution, participants are asked to “think aloud” (Birns et al., 2002), capturing their cognitive processes and decision-making logic throughout the task (see Figure 1).

### 3.2 Experiment setup

#### 3.2.1 Questionnaire and interview

Through in-depth interviews, we obtained information on designers’ preparation and implementation processes in their work, establishing an empirical foundation for the study. The questionnaire is designed based on the design brief creation process and core elements discussed in Chapter 2, aiming to bridge the gap between theory and practice. The primary goal is to identify and analyze the differences and connections between actual behaviors and theoretical concepts. Each designer has unique work habits based on their experience and the specific demands and constraints of their projects. Therefore, we need to analyze each case specifically, deeply understanding and recording designers’ real behaviors and thoughts to better comprehend the challenges and opportunities they face and to avoid discrepancies between theory and practice.

This questionnaire design is divided into two phases and seven sub-sections: Basic Information, Design Brief Process, Challenges, and Improvement Suggestions. Each section aims to collect specific information on how designers adapt to and handle various project requirements and constraints (Appendix for questionnaire content).

##### 3.2.1.1 Phase one

“As Is” Understanding the current state of design brief creation by designers.

- Basic Information: Collect background information about the designers, including their experience, expertise, and types of previous projects.
- Design Brief Process: Focus on understanding how designers develop and implement their design strategies based on the design brief.
- Challenges: Explore specific problems and difficulties designers encounter while implementing design strategies.
- Improvements: Gather designers’ specific suggestions and strategies for improving the design process and overcoming challenges.

##### 3.2.1.2 Phase two

“To Be” understanding designers’ experiences using AI tools to create design briefs.

- Experience of using AI to create design briefs: collect designers’ subjective experiences of using AI in the current project.
- Potential and challenges of AI: explore the potential and shortcomings of AI in the workflow.
- Expectations for AI: gather designers’ expectations for the future of AI-assisted workflows.

#### 3.2.2 User experience map

We use user experience maps as an evaluation tool to visually represent user behaviors during the process. Based on previous literature research on “design brief creation,” the designers’ workflow can be divided into three key stages:

##### 3.2.2.1 Prepare stage

Designers communicate with clients to clarify project goals, conduct market research, understand market trends and user needs, set clear design objectives, and assess the project’s time and resource requirements.

##### 3.2.2.2 Doing stage

The focus shifts to generating design concepts, creating initial designs or prototypes, and iterating and optimizing based on client feedback.

##### 3.2.2.3 After stage

Designers refine the design details, prepare and submit the complete design files, collaboratively evaluate the project outcomes with the client, ensure the project is successfully completed, and provide ongoing design support.

**Figure 2:**
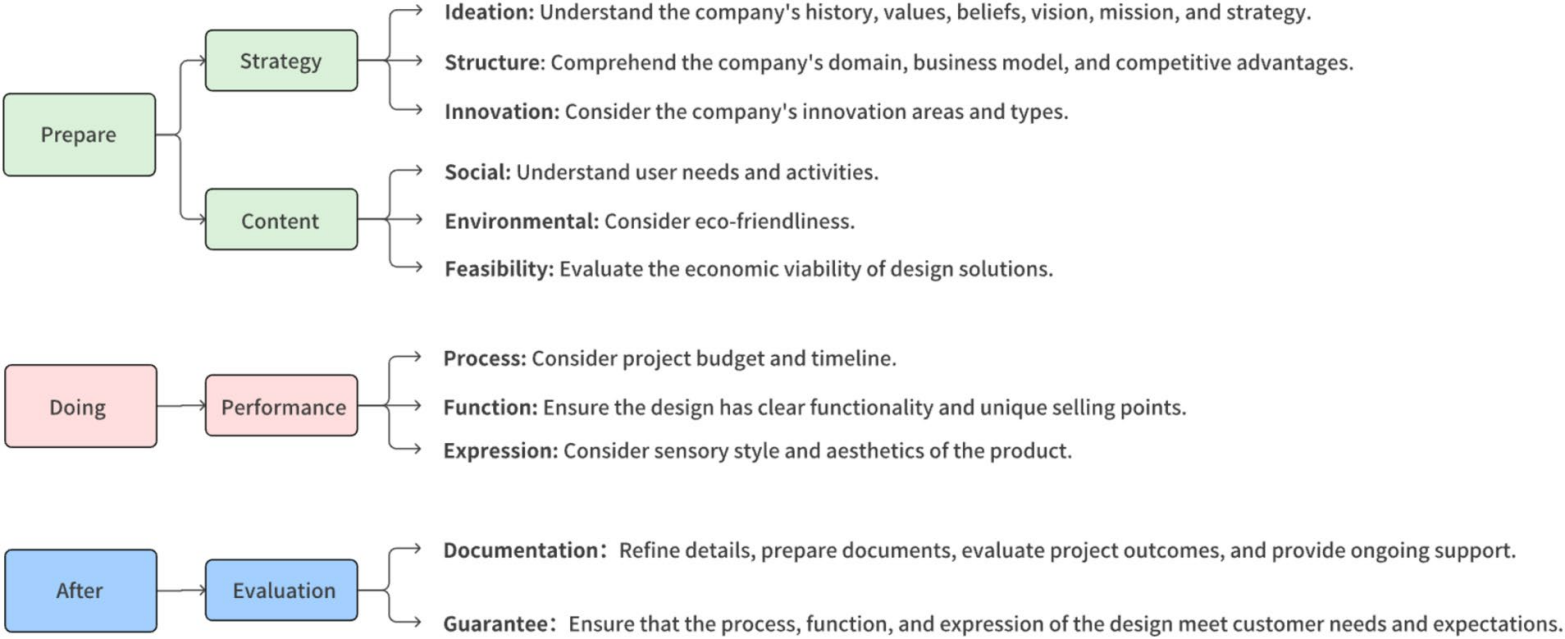
Experience map segmentation based on design brief.

These three stages form the complete process of creating a design brief, ensuring meticulous execution and detailed consideration at each project phase. The table below outlines the basic framework of this experiment, providing a structured reference for the study (see Figure 2).

### 3.3 Task setting

To ensure controlled experimental conditions, we constructed a virtual project and defined a series of related tasks. Given the complexity of project management and setting project direction and goals, we simplified this process while maintaining its practical relevance. The task design focused on key tasks within the Design Brief process and integrated elements from user experience maps. It encompassed the core components of user experience maps, divided into three phases with a total of 12 sub-tasks. Specifically, the initial phase included understanding the target company’s background, market analysis, exploration of innovation directions, user and market research, and economic feasibility assessment. The mid-phase involved budget and timeline management, functionality definition and optimization, and concept and style design. The final phase covered refining design details, preparing design documents, estimating project outcomes, and providing follow-up support and services. This design aims to visualize the execution process of the Design Brief, laying the foundation for subsequent evaluations of designers’ performance.

We selected a typical online game community platform project for this study, primarily due to its complex user interaction requirements and high market competitiveness. Such projects often require designers to balance user experience, functionality, and market demands, making it a representative case. Based on prior experience, the Design Brief for this type of project generally requires two designers working over a two-day period. To streamline the experiment, we excluded tasks related to innovation exploration and design document preparation, ensuring the experiment’s simplicity. ChatGPT 4.0 was chosen as the testing tool due to its intuitive conversational interface and low learning curve, allowing participants to complete the tasks without additional training, which ensured the smooth progression of the experiment, and the reliability of the data collected.

Additionally, we invited a project expert and used a commonly adopted project proposal evaluation system for third-party assessment: operability, comprehensibility, and accuracy. Operability checks if the actions in the document can achieve the project goals; comprehensibility evaluates whether the information is easy to understand, affecting communication efficiency; accuracy ensures all actions are progressing towards goal achievement. Finally, we visualized the testers’ stages, behaviors, touchpoints, feelings, pain points, and opportunities during the Design Brief creation process to provide a clear understanding of user behavior and process experience.

## 4 Results

We conducted interviews with 8 design experts, 5 of whom have a background in UX design and 3 in visual design. All participants had experience using AI tools in their work. These experts come from various business teams within their organizations, representing product areas such as automotive systems, gaming, social media, e-commerce, short-form video, and augmented reality. The rationale for selecting these participants was to ensure broad coverage of different fields while focusing on a common professional role.

Currently, there are no mature and comprehensive application standards for integrating AI into workflow processes as a new corporate work paradigm. Despite this, we observed a 100% AI usage rate among the designers, although the degree of reliance varied. In our sample, two designers exhibited high usage rates of AI tools, while the other six showed lower dependence. Nonetheless, all designers utilized AI to optimize and accelerate the processing of design briefs in their daily work (see Table 3).

**Table 3:** Designer status.

Working	Business type	Skill background	Frequency of daily use of AI tools	Common AI tools	Used AI Tools
Designer 1	5 years	Automobile user experience	UX Designer	High	ChatGPT
Designer 2	7 years	Game experience design	UX Designer	Medium	ChatGPT, Midjourney
Designer 3	9 years	Internet social experience design	UX Designer	Low	ChatGPT, Stable Defusion, Other
Designer 4	11 years	E-commerce experience design	Graphic Designer	Medium	ChatGPT
Designer 5	10 years	E-commerce experience design	UX Designer	Medium	ChatGPT
Designer 6	8 years	Short video platform experience design	Graphic Designer	High	ChatGPT, Stable Defusion, Midjourney
Designer 7	10 years	E-commerce experience design	Graphic Designer	Low	ChatGPT, Stable Defusion
Designer 8	9 years	AR experience design	UX Designer	Medium	ChatGPT

### 4.1 The creation of the traditional design brief

The accuracy of the design brief is directly linked to project timelines and costs. Below are the key points of interest and factors affecting the creation of design briefs as highlighted by the interviewed designers (see Table 4).

**Table 4:** Design brief production elements and executor concerns.

Key elements	Description	Requirements
Clear goals	The correct goals can lay the foundation for the active promotion of the entire project.	Accuracy
	Vague goals can lead to confusion in team management.	Accuracy
Functional scope	The functional scope involves the cost estimation of the project, which will affect the allocation of manpower and time.	Accuracy
	Collaboration and scheduling affect all design functions.	Efficiency
	Evaluate the scope to judge the workload	Efficiency
Historical data and documents	Understanding the past information of the demand side can correct goals and avoid repeated mistakes.	Accuracy
	Have overall control over the development of the current project.	Accuracy
Stakeholder communication	Maintain consistency among all parties regarding project objectives.	Accuracy
	Reduce the loss of information transmission.	Efficiency
	Improve project execution efficiency.	Efficiency
	Actively communicate to ease conflicts of interest.	Accuracy
End users	Always keep its design goals in mind	Accuracy
	Improve the mining of correct requirements	Accuracy
Known constraints	Can strengthen control over team resources	Efficiency
	Correct task boundaries to prevent resource waste	Accuracy
Rapidly verifiable prototype	Used to quickly report or persuade stakeholders in non-design functions	Efficiency
	Improve the production efficiency of design introduction documents	Efficiency

In project management, clear objectives, functional scope, historical data, and documentation are crucial for project success. In efficiency-driven enterprises, accuracy and efficiency are the main metrics for evaluating team performance. As shown in Table 4, accuracy ensures that the project progresses in the right direction, accounting for 59%, while efficiency involves cost control, resource allocation, and team collaboration, accounting for 41%. These influencing factors require designers to make repeated adjustments, with the core goal being “accurate goal setting” and “reasonable allocation of personnel resources”.

Designers need a method that is both accurate and efficient to optimize their workflows. Even if this method is not perfect, if it can save time and effort, it is worth considering. In this regard, AI tools offer a potential solution. Designers have expressed mixed evaluations of current AI tools, such as “ChatGPT helps organize tasks,” “significantly saves time,” and “AI-provided information needs careful handling as it occasionally contains inaccuracies that require verification.” These insights reveal both the effectiveness and limitations of AI tools.

To further understand the specific impact of AI tools on designers’ work, we will explore the operation and effectiveness of AI in practice in the second round of testing. This test aims to provide feedback and suggestions for the future development and use of AI tools. Through interviews with designers and documentation of actual work scenarios, we hope to gather more empirical data on how AI tools can improve designers’ work efficiency and accuracy.

### 4.2 Execution of specified projects without the use of AI

We focused on a medium-scale virtual project, the “Gaming Community” platform design (project description in the Appendix), which is considered a comprehensive project in actual work. During the experiment, we observed that designers met expectations in terms of operation time, task understanding, and initial background comprehension. The average time to understand the project background was 21 min, including the strategic and content sections. Participants quickly grasped the project background and task requirements. As the client, we provided necessary explanations and guidance to ensure designers clearly understood the task goals.

In the experiment, we found that designers needed to confirm the goals an average of 3.2 times. The average time for confirmation, cost estimation, resource allocation, and final document output was 72 min. To reduce fatigue effects from long-duration testing and alleviate task pressure, we divided the task execution into two phases, with the final document output task scheduled for the second phase.

After one round of testing, we recorded all designers’ behaviors and feelings during the tasks and used a 5-point evaluation system. The evaluation consisted of two parts: the first part assessed designers’ experiences in creating design briefs for the virtual project, focusing on how the information provided impacted task execution; the second part involved third-party evaluation of the final output reports. We scored nine tasks based on designers’ subjective experiences. The results showed lower scores for budget and project outcome evaluation when assessed solely by human evaluation, while higher scores were given for functionality clarity and subsequent support services.

### 4.3 Execution of specified projects using AI

One week after the first phase of testing, we invited the same eight designers for a second round of testing. This time, designers were required to use AI tools throughout the entire task. Designers first conducted the task test based on the documents we provided and then participated in interviews to share their operational experiences.

In terms of operation time, the average time for this test was 37 min, a reduction of 48.6% from the previous round, demonstrating significant effectiveness. Subjectively, designers’ overall evaluations improved, with positive feedback such as “The AI-generated report framework is very complete,” “It can generate content I had not thought of,” and “It can search for information according to my requirements.” There were also suggestions for improvement, such as “The authenticity of the output content needs further verification,” “The content is lengthy and needs manual correction,” and “It would be better if there were graphical outputs”.

Through the same task evaluations, we found that most operational experiences and subjective perceptions significantly improved with AI assistance. Specifically, designers’ average scores improved by 13.2% in understanding the target company’s background, 18.8% in market analysis, 5.3% in user and market research, 16.7% in economic feasibility assessment, 20.0% in budget and schedule management, 12.5% in functionality clarity and optimization, 7.1% in concept and style design, 7.1% in project outcome estimation, and 12.5% in follow-up support and services. These data illustrate the practical benefits and potential value of AI tools in designers’ daily work (see Table 5).

**Table 5:** Comparison of two tests.

No.	Task	Average score without using AI	Average score using AI	Difference
1	Understand the background of the target company (customer)	3.8	4.3	↑ 13.2%
2	Market analysis	3.2	3.8	↑ 18.8%
4	User and market research	3.8	4.0	↑ 5.3%
5	Economic feasibility assessment	3	3.5	↑ 16.7%
6	Budget and schedule management	2.5	3	↑ 20.0%
7	Function clarification and optimization	4	4.5	↑ 12.5%
8	Concept and style design	2.8	3	↑ 7.1%
11	Estimation of project results	2.8	3	↑ 7.1%
12	Follow-up support and services	4	4.5	↑ 12.5%

**Figure 3:**
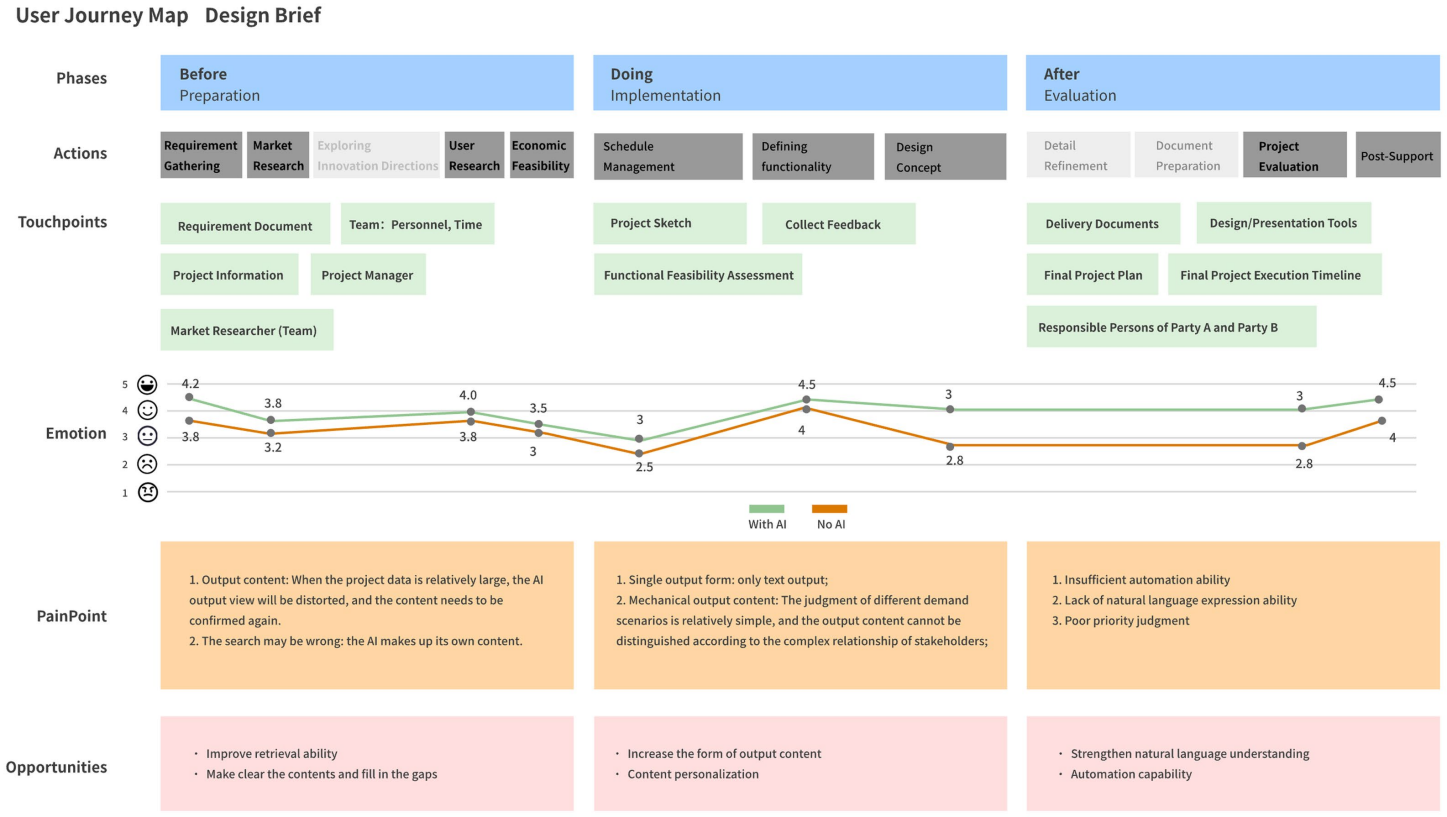
User experience map of actions and feedback from designers during the two tests.

We visualized and organized the designers’ operations and feedback from the second round of interviews into a user experience map. The figure below summarizes the designers’ usage experiences, identifies pain points with AI tools, and outlines potential future opportunities (see Figure 3).

In the “Before stages” of the process, designers primarily faced issues with the accuracy of AI tool outputs when handling large amounts of project data. For example, when project data is extensive, the AI-generated viewpoints may be distorted, requiring designers to verify and correct them. Additionally, AI sometimes fabricates content, increasing the burden on designers to ensure accuracy.

In the “Doing stage,” designers noted that AI tool outputs were relatively uniform, primarily limited to textual content. This restricted the designers’ flexibility in addressing diverse needs. Furthermore, AI-generated content appeared mechanical, lacking the ability to finely distinguish and personalize outputs for different scenarios. The AI’s insufficient automation capabilities, inadequate natural language expression, and poor prioritization in complex tasks further limited the efficiency and effectiveness of AI tools in designers’ daily work.

From an opportunity perspective, early-stage feedback from task tests indicated that designers believe AI tools can enhance their practical value by improving retrieval capabilities and filling content gaps. Mid-stage feedback suggested that expanding the types and personalization of outputs should be a focus for AI tool optimization. For instance, ChatGPT could list detailed feasibility options and provide illustrations based on requirements, surpassing designers’ expectations with comprehensive solutions and complete details. Enhancing automation capabilities is also a key improvement area desired by designers.

Due to the organization of numerous tasks through AI’s general knowledge base, designers saved a significant amount of time. Tasks that previously required separate resource requests, such as business canvases, user personas, and SWOT analyses, could now be completed quickly. The extraction of this content relied on designers’ knowledge systems, using the following prompts when making requests to the AI:

“Based on the above project background, please analyze the value proposition of the gaming community using the business canvas logic structure.”

“This value proposition is too broad. Please reorganize the content from the perspectives of channel diversity and different types of customer segmentation.”

“Please segment the target users and organize them in the format of user personas.”

“User type 2 encounters unclear goals when browsing the community, leading to quick attrition. Please develop an anti-attrition strategy for this situation.”

“Based on the above project information, product channels, user scale, and stakeholder relationships, summarize a SWOT analysis.”

We observed that the role of designers in their work has shifted, with AI becoming the “contractor,” while designers have transitioned from executors to the “client” of the project.

**Figure 4:**
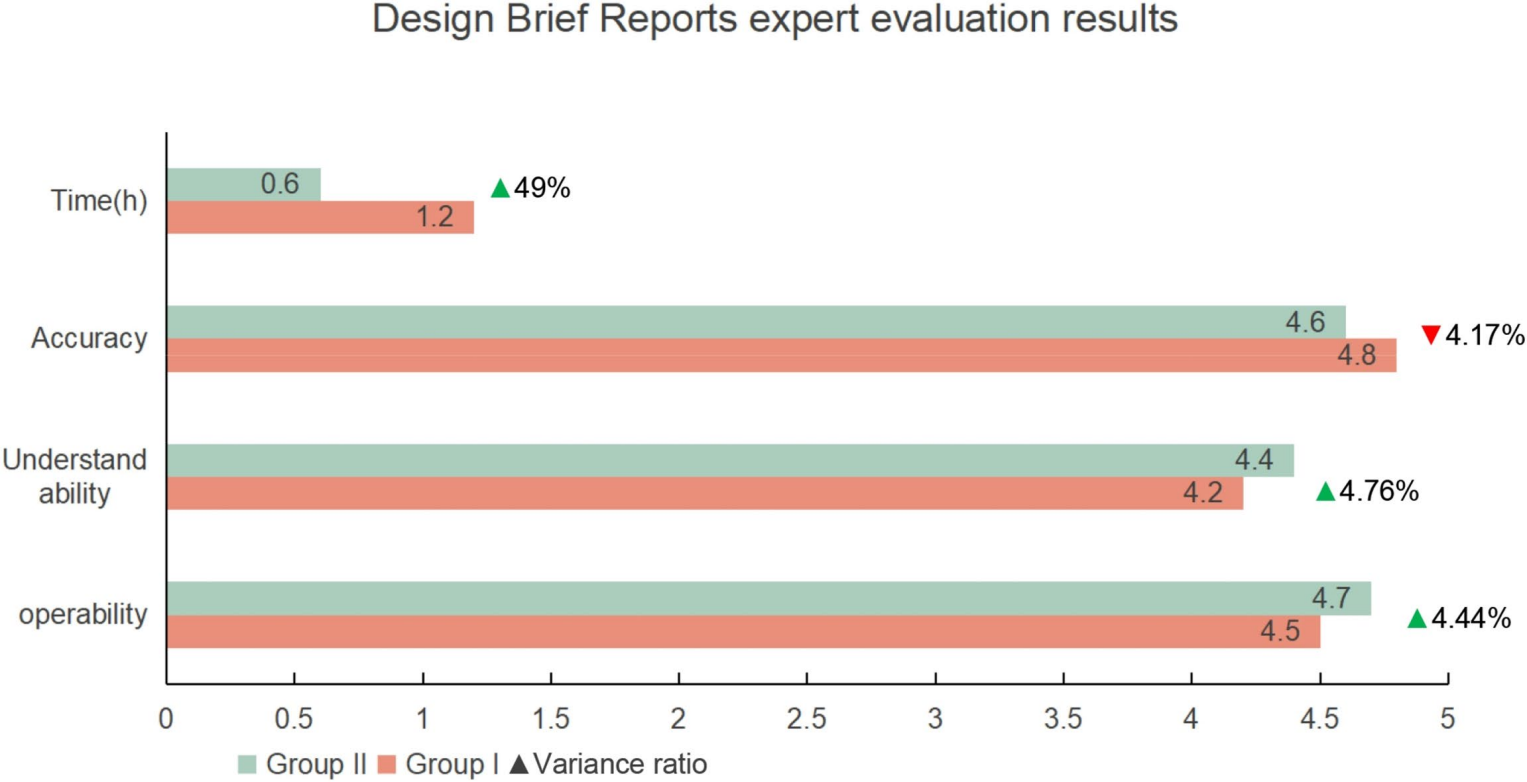
Comparison of third-party evaluation data.

After two rounds of testing, third-party experts to evaluate the design briefs prepared by the designers, assessing them based on operability, understandability, and accuracy. The evaluation results showed improvements in operability and understandability across the two rounds, but a slight decline in accuracy, with no significant overall difference. AI demonstrated a more comprehensive analysis in task and requirement matching. For instance, in the “workload allocation” task, ChatGPT, under the operator’s guidance, quickly provided three different plans and weighed their pros and cons. In contrast, manual evaluation mainly relied on the designer’s personal experience, which sometimes led to incomplete considerations (see Figure 4).

Compares two groups (Group I without AI tools and Group II with AI tools) across four criteria: Time (hours), Accuracy, Understandability, and Operability. Group II completed tasks significantly faster, reducing time by 49% (0.6 vs. 1.2 h). Understandability and operability improved by 4.76 and 4.44%, respectively, (4.4 vs. 4.2; 4.7 vs. 4.5). However, accuracy slightly decreased by 4.17% (4.6 vs. 4.8). These findings suggest that AI tools enhance efficiency, understandability, and operability, with a minor decline in accuracy.

Due to time constraints, AI’s answers were more comprehensive, resulting in higher scores for operability and understandability. However, the content generated by ChatGPT tended to be lengthy and, without an internet connection, could lead to distorted information, making it less accurate than manual evaluation.

## 5 Discussion

This study, through qualitative interviews and comparative experiments, explored how AI tools transform the process of creating design briefs by designers in corporate settings and their impact on work outcomes. The results indicate that using AI tools significantly enhances designers’ operational experience and satisfaction in most tasks, as visually demonstrated by the user experience map.

The integration of AI has the potential to revolutionize overall corporate workflows, particularly in improving management efficiency and operational smoothness. The study found that while design briefs are a critical part of the corporate production process, there are still some uncontrollable factors in their creation, such as the subjectivity of requirement interpretation, which can lead to increased production costs and extended production times (Tang et al., 2005; Cooper, 1998; Petersen, 2009; Rudin, 2019). Although the use of ChatGPT brought convenience, it also revealed issues such as data distortion and dependency on the manner of questioning (Li et al., 2021). These findings provide valuable insights into how to effectively leverage AI tools.

### 5.1 Back to the research question

Returning to the research questions, we can possess definite answers:

RQ.1 Designers commonly face specific obstacles and concerns while formulating design briefs in their routine professional endeavors.

When creating design briefs, designers must invest significant effort in obtaining accurate information to write development documents. The research revealed that this information falls into two categories: one involves interpreting background knowledge, such as unfamiliar project domains, understanding technical gaps, and verifying data accuracy; the other involves interpreting human factors, such as communicating with complex stakeholders and coordinating human resources. Completing as many tasks as possible within a limited time while addressing unforeseen issues during implementation often requires continuous revisions to the design brief. This significantly increases the workload of the design team and ultimately leads to a substantial rise in project costs.

RQ.2 Designers adhere to certain criteria when utilizing ChatGPT to develop a design brief.

The experiment revealed that when designers use ChatGPT to create design briefs, they primarily focus on information retrieval, verification, analysis, communication, and decision-making. They leverage ChatGPT’s generative capabilities to quickly gather and organize information, using prompts to identify and correct potential issues, and conduct in-depth data analysis to better understand client needs. Time efficiency is also a crucial factor for designers. By delegating repetitive and tedious information organization tasks to AI, which can complete them in a specified format, designers are able to concentrate on making important decisions.

RQ.3 There are noticeable disparities in terms of efficiency, cost, accuracy, and demand-side satisfaction between the utilization of ChatGPT and conventional approaches in creating design brief.

The differences between using and not using AI tools are significant, as reflected in both subjective experiences and objective data. Overall operation time was reduced by 48.6%, and subjective ratings across the remaining 12 evaluation metrics showed noticeable improvement. Designers using ChatGPT to develop design briefs benefited from its efficiency in quickly generating a large volume of relevant content. ChatGPT provides instant feedback and data-driven decision suggestions, which enhances productivity.

However, despite ChatGPT’s ability to generate extensive content, its accuracy can sometimes be compromised, particularly when the generated content is lengthy or contains distorted information. This suggests that when using AI tools, it is necessary to validate and adjust the generated content to ensure its reliability and accuracy.

RQ.4 Integrating AI tools into the workflow brings about specific advantages and constraints at the company level.

The application of AI tools in workflow processes can offer several enterprise-level benefits, including improved management efficiency, optimized production processes, enhanced human-machine and cross-role collaboration, and increased employee capacity to complete tasks. However, there are also some limitations to consider. Concerns about information authenticity, content copyright issues, and potential job displacement raise significant attention and need to be addressed.

In addition to addressing the research questions mentioned above, we also identified issues such as the “application of AI thinking in enterprise workflows” and the “impact of AI applications on designers” during our investigation. The following summarizes our findings:

### 5.2 Design with AI thinking

#### 5.2.1 AI thinking in enterprise workflows

A design brief ensures that all project participants remain aligned with the initial objectives, avoiding deviations. In traditional methods, designers are often troubled by the collection of data, validation of requirements, and the integration and organization of vast amounts of information. Obtaining accurate information requires significant time and effort from designers (Phillips, 2012; Dewulf, 2020). Under time constraints, the documents produced may encounter various issues during execution, necessitating continuous revisions. This not only increases the workload but also leads to significant cost overruns for the project team. Jones and Askland (2012) suggest that creating a design brief requires openness and flexibility, allowing for exploration and integration. With the assistance of AI, designers now have more guidance and options, enhancing the creation process.

For corporate managers, the primary focus is on data related to production costs. Any tool that can improve efficiency and optimize production processes is worth their attention. Therefore, the integration of such tools could benefit not only the design brief stage but virtually all production processes. Current trends indicate that AI is not just a tool for enhancing efficiency but also a strategy that needs to be standardized and deeply integrated into production workflows. The key findings from this study for enterprises are as follows:

#### 5.2.2 Design management

##### 5.2.2.1 Cost management

During task execution, we found that many processes are fixed. Automating tasks, optimizing resource allocation, and reducing human errors, especially in data processing, can significantly improve cost management.

##### 5.2.2.2 Risk management

Unexpectedly, during testing, ChatGPT generated content that included risk-related suggestions based on existing data, such as market risks and personnel limitations. For example, it suggested, “This task is complex, and with the current personnel arrangement, there is a risk of delay in meeting the deadline.” After evaluation by design experts, it was confirmed that there was indeed a potential for delay.

##### 5.2.2.3 Expectation management

Analyzing customer needs is a crucial part of project execution. AI technology enables enterprises to more accurately understand and meet customer needs and expectations. For instance, by analyzing market data and user feedback, AI can predict market trends and customer demands, helping businesses provide more personalized products or services.

#### 5.2.3 Multi-role collaboration

##### 5.2.3.1 Human-computer collaboration

The integration of AI prompts a reevaluation of the collaborative relationship between humans and computers. While computers can handle large volumes of data and repetitive tasks, designers possess unique strengths in creativity, strategic thinking, and emotional interaction. Effective human-computer collaboration can not only enhance work efficiency but also allow designers to focus more on innovation and high-level decision-making.

##### 5.2.3.2 Role collaboration

Different functions often incur significant time costs when communicating due to differences in knowledge systems. AI can bridge these cognitive gaps between roles. For example, through AI analysis, users can more accurately predict project timelines and resource needs, leading to better task allocation and team management.

### 5.3 The impact of AI tools on designers

In the results analysis of the previous section, we clearly observed the significant impact of AI tools on improving designers’ work efficiency. Human-Computer Interaction (HCI) primarily focuses on the interaction between humans and information. With the integration of AI, many information interaction tasks have been automated, prompting the emergence of the more advanced concept of “Human-Centered AI” (HCAI) (Fui-Hoon Nah et al., 2023). In this context, AI is not just a simple tool but a whole new way of thinking. Mastering and skillfully utilizing this AI mindset will become crucial for future product development and project planning. From our interviews and test tasks, we identified the following five points reflecting designers’ perspectives on AI tools when using ChatGPT.

#### 5.3.1 Positive impact

##### 5.3.1.1 Information retrieval

Traditionally, establishing an information framework in the early stages required extensive time to understand background knowledge. ChatGPT’s generative capabilities allow it to manage relevant content directly and serve as a search or retrieval tool. For example, by inputting a keyword, AI can return a large amount of related content in a short time, helping designers quickly gather and organize information.

##### 5.3.1.2 Verification

AI can efficiently process vast amounts of data and information, providing multiple feedback options for the user. This instant feedback helps designers identify and correct potential issues early, thus avoiding extensive modifications later.

##### 5.3.1.3 Analysis

AI tools can perform in-depth analysis of large amounts of user data, assisting designers in better understanding customer needs.

##### 5.3.1.4 Communication

AI breaks down knowledge barriers, helping designers communicate more effectively with team members, clients, and stakeholders.

##### 5.3.1.5 Decision-making

AI can offer data-supported decision-making suggestions to designers. Based on extensive data analysis, AI can predict the possible outcomes of a design decision, helping designers make more informed and substantiated choices.

From the feedback above, AI technology enhances designers’ efficiency through quick information retrieval, instant feedback, in-depth data analysis, and decision-making suggestions. It also facilitates communication between designers, teams, and clients. However, these benefits come with challenges, such as concerns about information authenticity, copyright issues, and potential job displacement, leading to professional anxiety.

#### 5.3.2 Facing challenges

##### 5.3.2.1 Misinformation

Designers generally expressed concerns about the authenticity of AI-generated information. Errors may arise from biases in training data, algorithm limitations, or other factors. Therefore, designers should maintain critical thinking and verify and review the information provided by AI tools.

##### 5.3.2.2 Copyright and privacy issues

This study employed ChatGPT as the primary tool, and given its text-based output, concerns regarding copyright and privacy were relatively minimal. However, when it comes to multimedia content such as images, videos, or audio, copyright issues cannot be ignored. Although the interviewees did not explore these issues in depth, they emphasized the importance of “respecting copyright and ensuring data security, adhering to commercial or industry standards to avoid disputes.” In commercial applications, companies typically train models using proprietary databases, such as self-developed large models, and label content as “AI-generated” to avoid copyright conflicts. At the same time, the design industry urgently needs to establish clear regulations to define the ownership and usage rights of AI-generated content. Companies should also implement internal policies to properly address copyright issues when using AI tools, to mitigate legal risks. Besides copyright concerns, AI also faces challenges due to the“black box”nature, which leads to a lack of transparency in decision-making processes (Rudin, 2019). Ongoing debates surrounding ethics, privacy, data, and copyright remain unresolved, causing the public to approach AI-generated commercial content with caution, which has, to some extent, shaped designers’perspectives on AI tools (Zhang et al., 2024).

##### 5.3.2.3 Professional identity

Our preliminary research found that many companies are reevaluating their workflows, particularly in design and software development departments, which are profoundly affected by AI. For instance, in the graphic design field, approximately 50% of work is now done by graphic AI tools such as Midjourney and Stable Diffusion. Similarly, tools like Co-Pilot assist developers in the software development field. While these AI tools improve productivity, they may also lead to job displacement, causing professional anxiety among employees.

**Figure 5:**
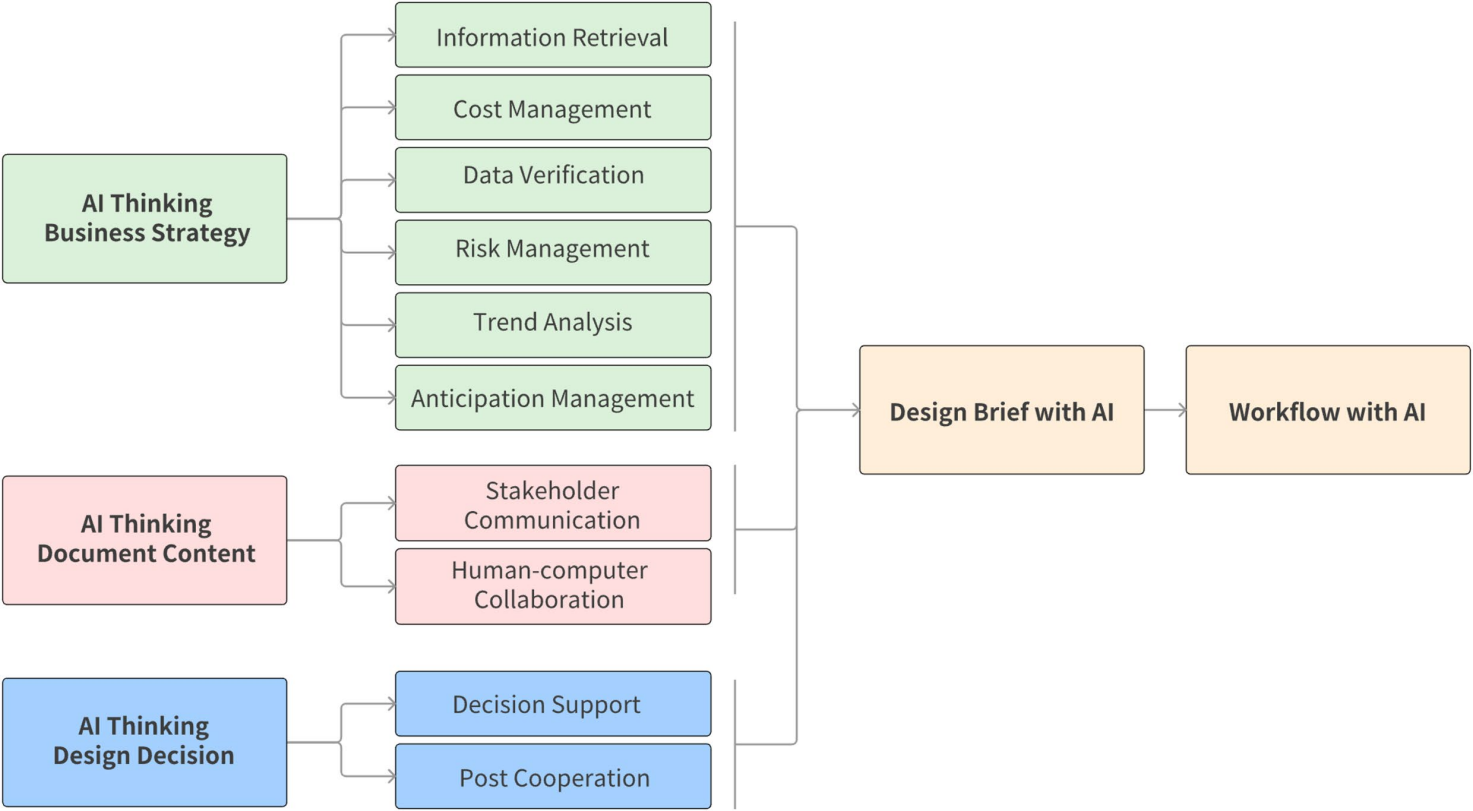
AI thinking in workflow.

The application of AI is not limited to the creation of design briefs. AI can play a supportive role throughout the construction and implementation of entire workflows, helping enterprises achieve their business objectives while also assisting employees in completing their tasks more effectively. As illustrated below, we integrate AI thinking into key processes such as business strategy formulation, content generation for documentation, and design decision-making, identifying the specific tasks that AI can undertake in these areas (see Figure 5).

## 6 Conclusion

This study explores how the AI tool ChatGPT affects the process of creating design briefs by designers in a corporate setting and evaluates the impact of using versus not using AI tools on their work. The experiment was conducted in two phases: the first phase without using AI tools, and the second phase requiring designers to use AI tools throughout the process. The results showed that the operational experience and subjective perceptions of most tasks significantly improved after using AI tools. The entire testing process was visually compared using a user experience map. The study also examined the application of AI in overall corporate workflows, highlighting its potential value in enhancing management efficiency and operational smoothness.

### 6.1 Contributions

In contrast to previous studies, we closely observed designers’ behaviors and thoughts through experiments when using AI tools. The new tools demonstrated significant improvements in work efficiency, while also revealing other issues. Additionally, using user experience maps for visual comparison is a novel approach that offers practical tools for future research and implementation. Furthermore, the study shows that AI can seamlessly integrate into corporate workflows, enhancing overall efficiency and achieving business objectives.

### 6.2 Limitations of the study

The limitations of this study are also evident, including reliance on qualitative research methods and a small sample size, which may restrict the breadth of the findings. The participants were primarily experienced designers, so the results may have limited applicability to novice or less experienced designers. Additionally, the experimental setup based on virtual projects and internal tests may not fully replicate real-world working conditions, necessitating caution when applying the findings in practice.

### 6.3 Future research directions

This study focused on creating design briefs for corporate projects and achieved the expected results, demonstrating the positive impact of AI tools. Through communication with various industry experts, we identified more interesting research topics.

#### 6.3.1 Next, generation designer identity

AI differs from traditional efficiency tools, and as artificial general intelligence (AGI) develops, the boundaries between designers and other roles are becoming increasingly blurred. For instance, visual designers may directly engage in programming, while programmers may design UI interfaces. As a result, the identity and position of designers need to be reexamined. Future research could explore how designers adapt to these changes and their evolving interactions with AI technologies.

#### 6.3.2 AI automation and standardization

Our research revealed significant differences in how designers use AI tools, leading to inconsistent output. Future studies could focus on establishing standardized processes to optimize AI tool usage, particularly by setting prompt structures and parameters to guide AI tasks. This would enhance work efficiency and promote the automation of the design process.

#### 6.3.3 AI and design career confidence

This study also found that AI tools significantly affect designers’ professional outlooks at different experience levels. Junior, mid-level, and senior designers perceive and use AI in distinct ways, but there is a lack of systematic quantitative research on this topic. Future research should delve deeper into how AI impacts designers’ professional confidence and career development to better understand AI’s long-term effects on the design industry.

#### 6.3.4 Longitudinal research and industry comparisons

Future studies could conduct longitudinal research to assess the long-term impact of AI on design roles or compare the application of AI across different industries. This would provide a more comprehensive understanding of AI’s profound influence on the design field and offer tailored strategies and recommendations for designers in various sectors.

## Data Availability

The original contributions presented in the study are included in the article/supplementary material, further inquiries can be directed to the corresponding author.
